# Supplementary material for: Quantifying persistence in the T‐cell signaling network using an optically controllable antigen receptor
Source: Mol Syst Biol. 2021 May 14;17(5):e10091. doi: 10.15252/msb.202010091 (PMC8120804; doi:10.15252/msb.202010091)
Supplement: Supplementary file 2 — Appendix [file MSB-17-e10091-s003.pdf]

Table of Contents

Figure S1: Light-induced dissociation of OptoCAR construct. 2
Figure S2: RT-qPCR data for experiments shown in Figure 5. 3
Figure S3: Vector construction and protein sequence of OptoCARs. 4

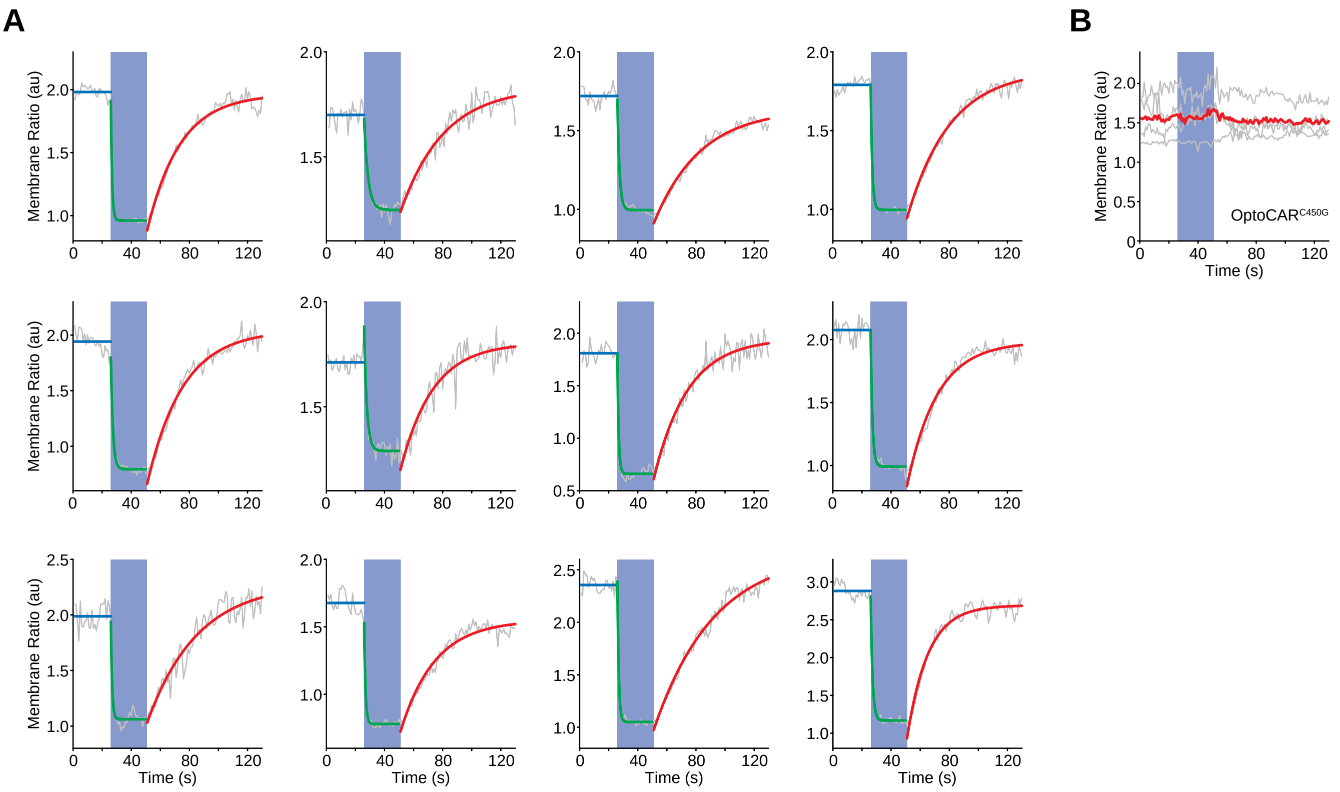

Figure S1. Light-induced dissociation of OptoCAR construct.

A The individual plots from the 12 analyzed cells used to prepare Figure 1E are shown without normalization. The ratio of membrane-bound intracellular part of OptoCAR compared to the the cytoplasm is quantified over time. Cells were illuminated after 25 seconds of imaging for 25 seconds to calculate dissociation rate (green) before returning to dark (signaling competent) state to calculate re-association rate (red line). These individual fits were used to calculate the mean rate parameters presented in the main text.

B The C450G variant of LOV2 was inserted into the OptoCAR and the equivalent experiment as in (A) was performed. No significant decrease in membrane fluorescence was observed with this variant, ruling out phototoxic effects as an explanation for the measured OptoCAR dynamics. Five cell traces are shown, with mean (n=5) presented as red line.

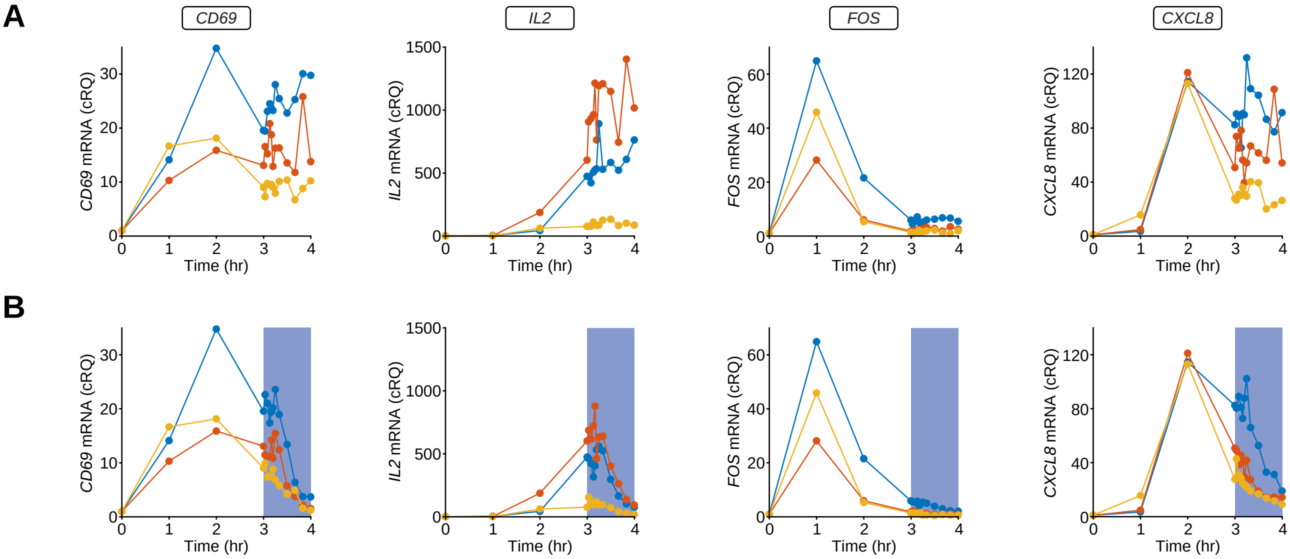

Figure S2. RT-qPCR data for experiments shown in Figure 5.

A The cRQ values for the RT-qPCR datasets maintained in the dark state are presented without additional scaling. The mRNA levels were calculated relative to the 0 min sample and then normalized to the geometric mean of *PGK1* and *GAPDH* mRNA (housekeeping genes) to control for variable cDNA quantity, forming the cRQ values. Each biological replicate is colored separately and the gene name for each dataset is given in the boxes above plots.

B Equivalent plots as in (A) but now showing the light-induced cessation of signaling at 3 hours, shown with the blue region.

A

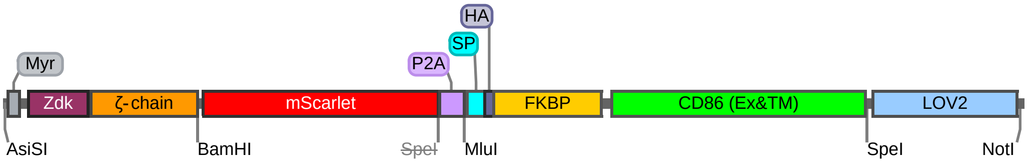

B

```
  1 MGCGCSSHPE DDGGSGGSGG SMVDNKFNKE KTRAGAEIHS LPNLNVEQKF AFIVSLFDDP SQSANLLAEA KKLNDAQAPK TSADAPAYQQ GQNQLYNELN 100
101 LGRREEYDVL DKRRGRDPEM GGKPQRRKNP QEGLYNELQK DKMAEAYSEI GMKGERRRGK GHDGLYQGLS TATKDTYDAL HMQALPPRDP PVATMVSKGE 200
201 AVIKEFMRFK VHMEGSMNGH EFEIEGEGEG RPYEGTQTAK LKVTKGGPLP FSWDILSPQF MYGSRAFTKH PADIPDYYKQ SFPEGFKWER VMNFEDGGAV 300
301 TVTQDTSLED GTLIYKVKLR GTNFPPDGPV MQKKTMGWEA STERLYPEDG VLKGDIKMAL RLKDGGRYLA DFKTTYKAKK PVQMPGAYNV DRKLDITSHN 400
401 EDYTVVEQYE RSEGRHSTGG MDELYKTSYQ GPGATNFSLL KQAGDVEENP GPTRMGVKVL FALICIAVAE AKYPYDVPDY AGVQVETISP GDGRTFPKRG 500
501 QTCVVHYTGM LEDGKKVDSS RDRNKPFKFM LGKQEVIRGW EEGVAQMSVG QRAKLTISPD YAYGATGHPG IIPPHATLVF DVELLKLEGG SGGSGGSLKI 600
601 QAYFNETADL PCQFANSQNQ SLSELVVFWQ DQENLVLNEV YLGKEKFDSV HSKYMGRTSF DSDSWTLRLH NLQIKDKGLY QCIIHHKKPT GMIRIHQMNS 700
701 ELSVLANFSQ PEIVPISNIT ENVYINLTCS SIHGYPEPKK MSVLLRTKNS TIEYDGIMQK SQDNVTELYD VSISLSVSFP DVTSNMTIFC ILETDKTRLL 800
801 SSPFSIELED PQPPPDHIPW ITAVLPTVII CVMVFCLILW KWKKKKRPRT SGGSGSLATT LERIEKNFVI TDPRLPDNPI IFASDSFLQL TEYSREEILG 900
901 RNCRFLQGPE TDRATVRKIR DAIDNQTEVT VQLINYTKSG KKFWNLFHLQ PMRDQKGDVQ YFIGVQLDGT EHVRDAAERE GVMLIKKTAE NIDEAAKEL* 999
```

C

```
pr1: 5′ gtagactagtggcggaagcggaagtttggctactacacttgaacgtattg 3′
pr2: 5′ ctactagcggccgctcaaagttcttttgccgcctcatc 3′
pr3: 5′ tagtaggcgatcgccaccatgggctgtggctgcagc 3′
pr4: 5′ cgccagggccctggtaactGgtcttgtacagctcgtccatgc 3′
pr5: 5′ gcatggacgagctgtacaagacCagttaccagggccctggcg 3′
pr6: 5′ tagtaggcgatcgccacctggtggataacaaattcaataaagaaaag 3′
pr7: 5′ ctactaggatcccgaggggcagggcc 3′
```

D

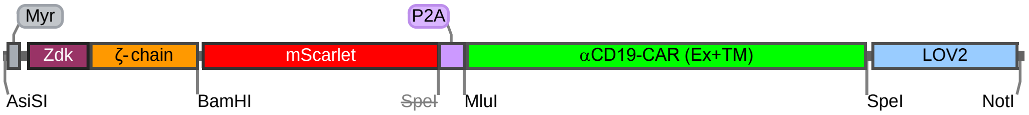

E

```
  1 MGCGCSSHPE DDGGSGGSGG SMVDNKFNKE KTRAGAEIHS LPNLNVEQKF AFIVSLFDDP SQSANLLAEA KKLNDAQAPK TSADAPAYQQ GQNQLYNELN 100
101 LGRREEYDVL DKRRGRDPEM GGKPQRRKNP QEGLYNELQK DKMAEAYSEI GMKGERRRGK GHDGLYQGLS TATKDTYDAL HMQALPPRDP PVATMVSKGE 200
201 AVIKEFMRFK VHMEGSMNGH EFEIEGEGEG RPYEGTQTAK LKVTKGGPLP FSWDILSPQF MYGSRAFTKH PADIPDYYKQ SFPEGFKWER VMNFEDGGAV 300
301 TVTQDTSLED GTLIYKVKLR GTNFPPDGPV MQKKTMGWEA STERLYPEDG VLKGDIKMAL RLKDGGRYLA DFKTTYKAKK PVQMPGAYNV DRKLDITSHN 400
401 EDYTVVEQYE RSEGRHSTGG MDELYKTSYQ GPGATNFSLL KQAGDVEENP GPTRMALPVT ALLLPLALLL HAARPDIQMT QTTSSLSASL GDRVTISCRA 500
501 SQDISKYLNW YQQKPDGTVK LLIYHTSRLH SGVPSRFSGS GSGTDYSLTI SNLEQEDIAT YFCQQGNTLP YTFGGGTKLE ITGGGGSGGG GSGGGGSEVK 600
601 LQESGPGLVA PSQSLSVTCT VSGVSLPDYG VSWIRQPPRK GLEWLGVIWG SETTYYNSAL KSRLTIIKDN SKSQVFLKMN SLQTDDTAIY YCAKHYYYGG 700
701 SYAMDYWGQG TSVTVSSTTT PAPRPPTPAP TIASQPLSLR PEACRPAAGG AVHTRGLDFA CDIYIWAPLA GTCGVLLLSL VITLYCKKKK RPRTSGGSGS 800
801 LATTLERIEK NFLITDPRLP DNPIIFASDS FLQLTEYSRE EILGRNCRFL QGPETDRATV RKIRDAIDNQ TEVTVQLINY TKSGKKFWNL FHLQPMRDQK 900
901 GDVQYFIGVQ LDGTEHVRDA AEREGVMLIK KTAENIDEAA KEL* 943
```

Figure S3. Vector construction and protein sequence of OptoCARs.

A Schematic showing the various components part of the OptoCAR construct as they are arranged in the lentiviral vector used to express the receptor in T cells. Relevant restriction sites are highlighted. Myr denotes myristoylation sequence and SP is signal peptide sequence.

B Protein sequence of OptoCAR, using colors from (A) to denote different regions. Point mutations in LOV2 domain used for OptoCAR variants are highlighted red.

C Oligonucleotide sequences used in construction of OptoCAR vectors.

D Schematic showing the various components part of the OptoCAR^{CD19} construct as they are arranged in the lentiviral vector used to express the receptor in T cells. Relevant restriction sites are highlighted. Myr denotes myristoylation sequence.

E Protein sequence of OptoCAR^{CD19}, using colors from (D) to denote different regions.
