# Supplementary material for: Quantifying persistence in the T‐cell signaling network using an optically controllable antigen receptor
Source: Mol Syst Biol. 2021 May 14;17(5):e10091. doi: 10.15252/msb.202010091 (PMC8120804; doi:10.15252/msb.202010091)
Supplement: Supplementary file 3 — Expanded View Figures PDF [file MSB-17-e10091-s005.pdf]

Expanded View Figures

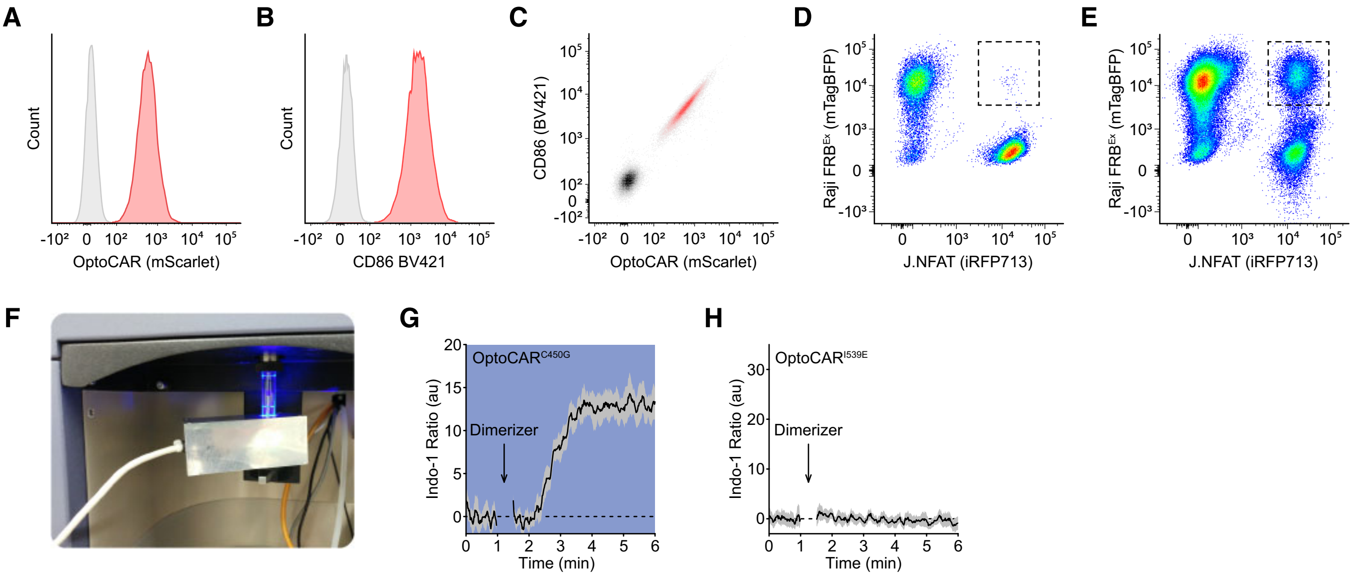

Figure EV1. Quantifying the intracellular Ca^{2+} flux in conjugated OptoCAR-T cells. Related to Fig 2.

A–C Flow cytometry data of Jurkat T cells either untransduced (gray) or expressing the OptoCAR construct (red) after lentiviral transduction. The distribution of mScarlet fluorophore genetically fused to OptoCAR is shown (A), along with surface staining for the receptor using an anti-CD86 antibody (B). The bivariate plot of these two distributions shows that they are extremely well-correlated (C), demonstrating mScarlet intensity is a good marker for OptoCAR cell surface expression.

D, E Conjugates formed between Jurkat (J.NFAT; iRFP713^{+}) and Raji-FRB^{Ex} cells (mTagBFP^{+}) can be directly gated on during acquisition of flow cytometry data. Simply mixing the two cell types together produces essentially no double-positive events (D), but allowing them to interact at high density at 37°C for 10 min creates a distinct and readily observable population of cell conjugates (E).

F Photograph of the custom-built illumination device that can expose the sample to blue light while maintaining it at 37°C on the flow cytometer.

G, H Repeating the Ca^{2+} flux assay described in the main text with a variant of the LOV2 domain in the OptoCAR that is unresponsive to blue light illumination (OptoCAR^{C450G}) drives increased intracellular [Ca^{2+}] independently from the illumination state of the sample (G). Bounded line shows mean ± SEM (n = 3). Conversely, the complementary mutation of the LOV2 domain that maintains it in the "light" state (OptoCAR^{I539E}) cannot drive signaling even in the dark (H). Bounded line shows mean ± SEM (n = 4).

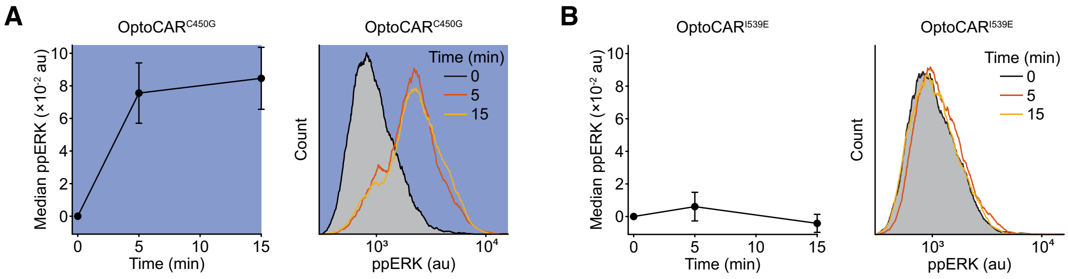

Figure EV2. Light-induced dissipation of ERK phosphorylation is signaling-dependent. Related to Fig 3.

A OptoCAR^{C450G}-T cells were conjugated as for the main experiments shown in Fig 3 but exposed to continuous illumination from dimerizer addition at 0 min for 15 min. Left panel shows the median ppERK intensity. Bars show mean ± SEM (n = 3). Right panel shows representative ppERK distributions from this control experiment.

B OptoCAR^{I539E}-T cells were conjugated as for the main experiments shown in Fig 3 and remained in the dark state from dimerizer addition at 0 min for 15 min. Left panel shows the median ppERK intensity. Bars show mean ± SEM (n = 3). Right panel shows representative ppERK distributions from this control experiment.

© 2021 The Authors

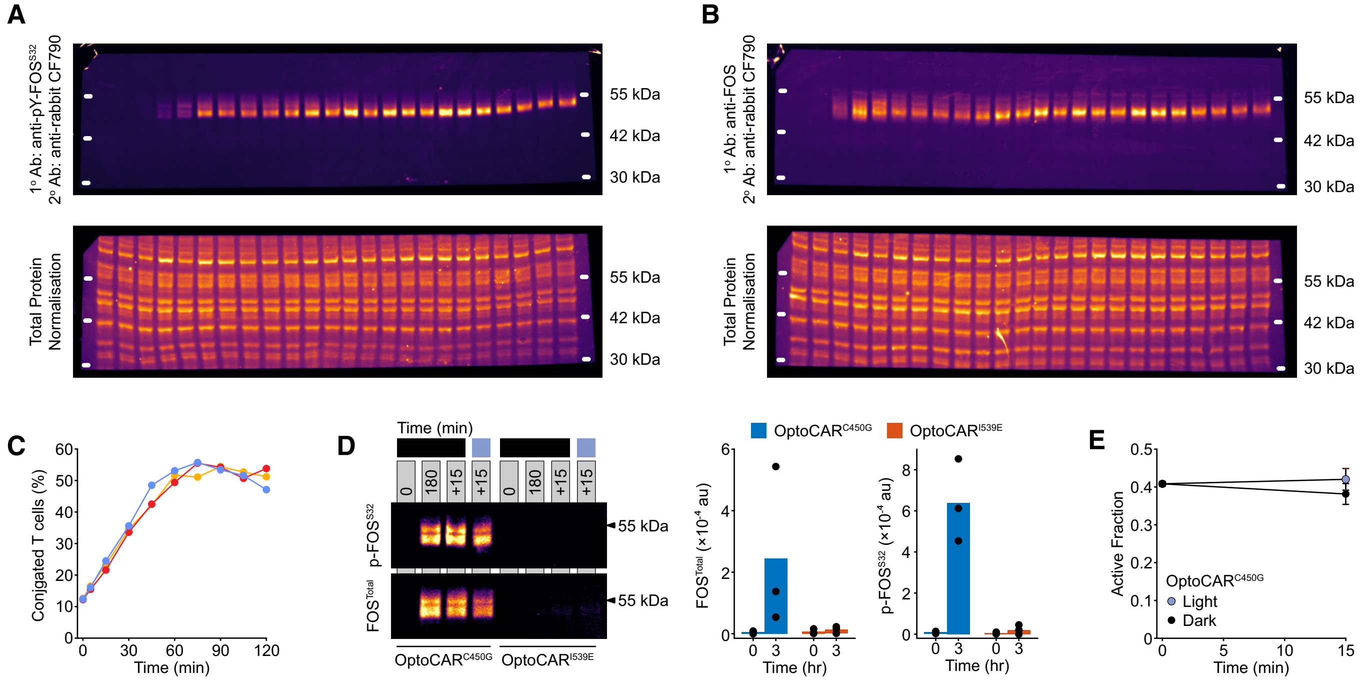

Figure EV3. Control experiments and uncropped Western blots for FOS activation datasets. Related to Fig 4.

A Uncropped fluorescent Western blots from data presented in Fig 4A. The accompanying total protein normalization (TPN) blot for this Western is also provided in the lower image. The TPN blot is a more robust means to account for variable sample loading by visualizing all proteins blotted from the gel, rather than just one or two representative proteins, such as GAPDH or tubulin. Molecular weight markers were determined from an additional fluorescence channel.

B Uncropped fluorescent Western blots from data presented in Fig 4B. The accompanying total protein normalization blot for this blot is also provided in the lower image. Molecular weight markers were determined from an additional fluorescence channel.

C The fraction of OptoCAR-T cells conjugated with Raji-FRB^{Ex} cells over time was measured by flow cytometry. Dimerizer was added to the cells at the start of experiment, which drives more stable conjugates. Each colored line represents a replicate test.

D An equivalent assay was run as that shown in Fig 4 but using either the OptoCAR^{C450G} or OptoCAR^{I539E} variants. Conjugated OptoCAR-T cells were activated with dimerizer, and samples were taken at 0 and 180 min. Samples were then either left in the dark state for a further 15 min are illuminated during this period. OptoCAR^{I539E} did not drive detectable levels of FOS expression as expected. Three independent replicates are presented in the right panel bar charts.

E Quantification of the active fraction from the experiments in (D) showed there was no significant effect of illumination on FOS activity. Bars show mean ± SEM (n = 3).

 ©2021 The Authors

Figure EV4. Downstream output from OptoCAR-T cells can be modulated by illumination. Related to Fig 5.

A The ability of illumination to modulate NFAT-mediated GFP expression or CD69 upregulation from OptoCAR-T cells was tested by either activating cells continuously in the dark (maximal output) or light state (denoted in boxes beside plots). Vehicle control (no dimerizer) was used to measure baseline expression. Plots show flow cytometry data with GFP or CD69 intensity against OptoCAR expression.

B The output expression of GFP or CD69 was measured after 24 h, with conjugated OptoCAR-T cells illuminated continuously with varying intensities of light. The expected decrease in output with light is observed, and the light intensity used for the experiments in the main text is denoted by arrows.

C The C450G (light-insensitive) and I539E (non-binding) LOV2 variants of OptoCAR-T cells were used to account for phototoxic effect of continuous illumination on output expression after 24 h. As expected, OptoCAR^{I539E}-T cells showed no response in either light or dark. OptoCAR^{C450G}-T cells were activated in both conditions, although a slight decrease in output was observed under continuous illumination, which we take as a measure of phototoxicity. All pulse-frequency modulated datasets in main text use constant total illumination time to account for this.

D Representative flow cytometry plots from datasets in (C) showing the minimal decrease in output observed for the activated OptoCAR^{C450G}-T cells variants under constant illumination.

E The mean of NFAT-mediated GFP expression from OptoCAR-T cell conjugates over time, acquired by confocal microscopy. Individual traces from 5 stitched regions of experimental images are shown in gray lines, with mean time course in black. Datasets have been corrected for background fluorescence and scaled relative to GFP expression after 24 h.

F Density plot of NFAT-mediated GFP or CD69 expression as a function of varying the length of a single-stimulus pulse. OptoCAR-T cell conjugates were activated in the dark (signaling-competent) state for a defined period before light-mediated disruption of signaling for the remainder of a 24-h period; cells were all assayed after this time. Plot is composed of 24 individual flow cytometry distributions.

G OptoCAR-T cells were activated in the dark state for 16 h and then either maintained in the dark (black), illuminated (blue), or cycloheximide added (yellow), to measure the degradation of GFP or CD69 surface expression with time. Samples were collected at different time points after the onset of the perturbation (0 h) and the two outputs measured by flow cytometry. Cycloheximide acutely inhibits protein synthesis, whereas illumination caused a delayed (~1-h) reduction in output, presumably due to the persistence of mRNA described in main text. However, both perturbations provided very similar estimates of output degradation, with rate constant provided in each panel. Bars show mean ± SEM (n = 4).

H A pseudo-time course of GFP and CD69 expression using the optoPlate, with illumination of one dataset after 6 h. All conjugated OptoCAR-T cells were activated by dimerizer addition at 0 h and output measured after 24 h. To simulate a time-course experiment, samples were illuminated at the start of assay until the prescribed time for activation, e.g., for the 8-h time point illumination occurred from 0 to 16 h and then signaling initiated in the dark state from 16 to 24 h. This allowed all 96 samples to be stained and measured at a single endpoint. The effective cessation of signaling at 6 h caused the output to plateau as anticipated, although this took a significant period of time due to requirements for protein folding and maturation. Bounded lines show mean ± SEM (n = 3).

I Combined plot of all datasets from Fig 6E and F but only over light interval times up to 15 min, with left panel showing GFP output and right showing CD69. No additional processing of the datasets has been performed.

J An equivalent experiment as in Fig 6E but now all output from datasets was measured after 12 h (and not 24 h). The sum of signal pulses was still 6 h, but this necessarily limited the duty cycles to range from 100 to 50%. Bars show mean ± SEM (n = 3) of biological replicates.

K An equivalent experiment as in Fig 6F but now all output from datasets was measured after 12 h (and not 24 h). The sum of signal pulses was still 6 h, but this necessarily limited the duty cycles to range from 100 to 50%. Bars show mean ± SEM (n = 3) of biological replicates.

© 2021 The Authors

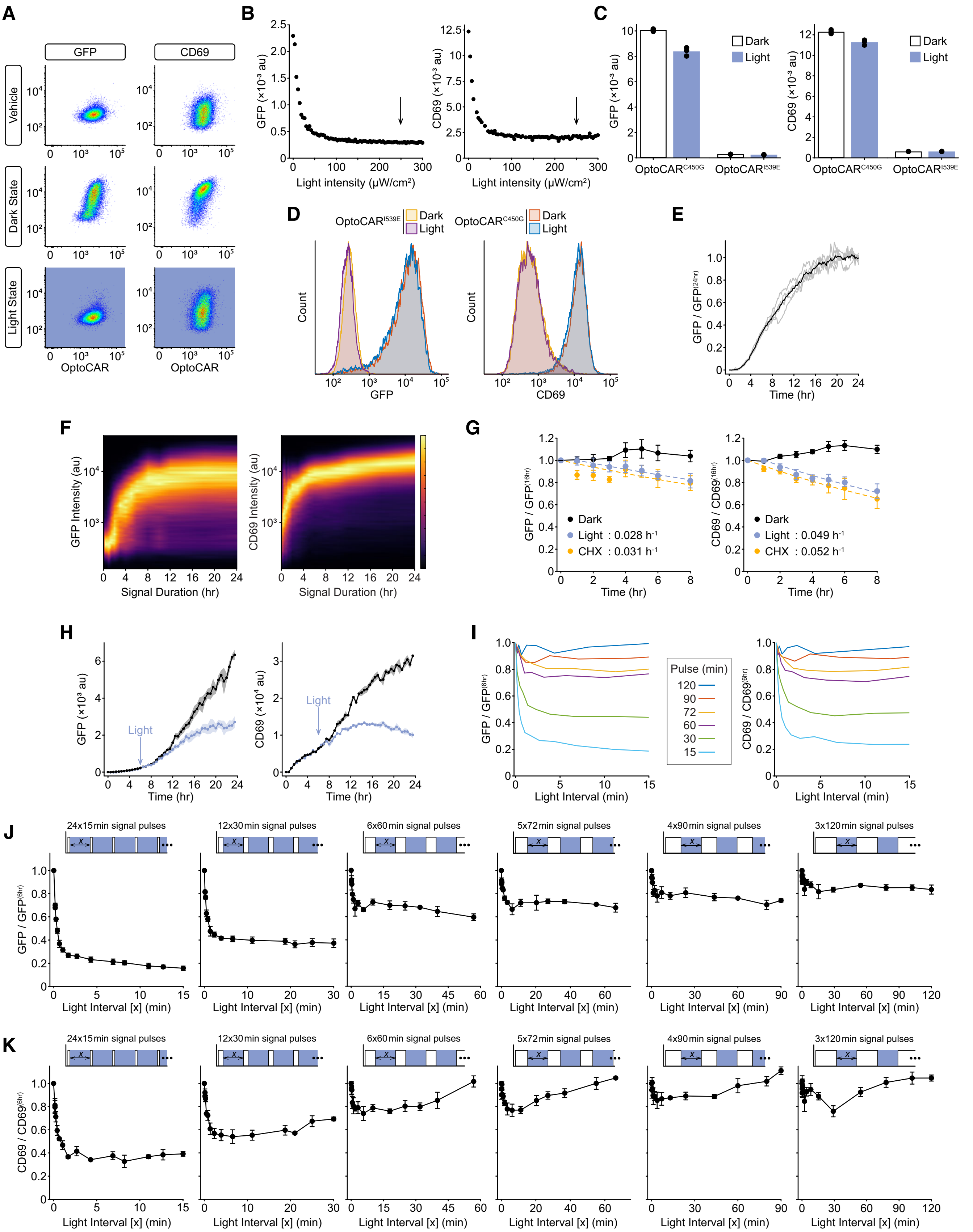

Figure EV4.

©2021 The Authors

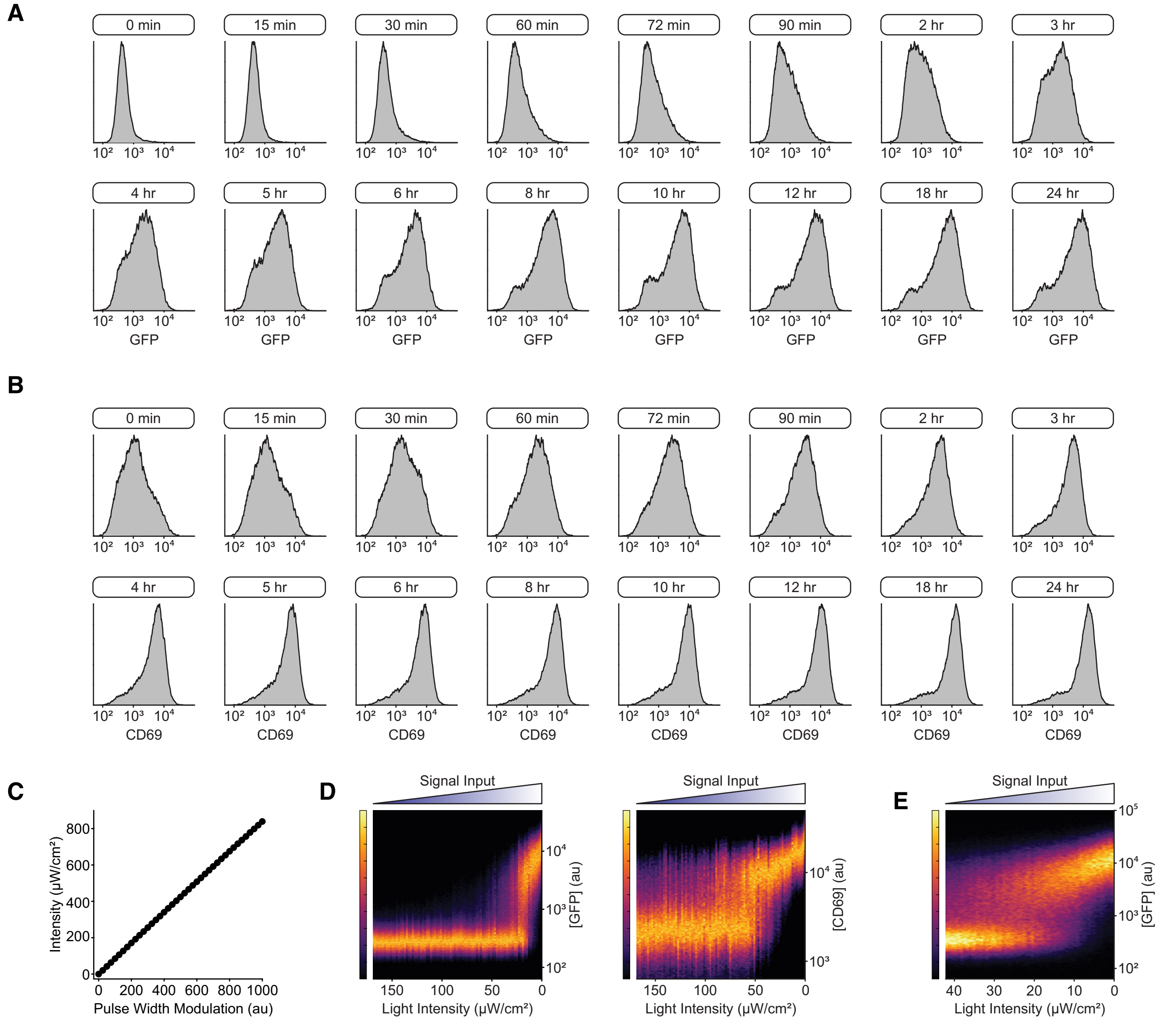

Figure EV5. Graded illumination of OptoCAR-T cells drives both analogue and digital outputs.

A Flow cytometry plots showing the distribution of NFAT-mediated GFP expression after different single-pulse lengths (denoted in boxes above plots), measured after 24 h. A subset of pulse lengths is shown, ranging from 15 min to 24 h. These distributions were used to build the density plot in Fig EV4F.

B Flow cytometry plots showing the distribution of CD69 upregulation after different single-pulse lengths (denoted in boxes above plots), measured after 24 h. A subset of pulse lengths is shown, ranging from 15 min to 24 h. These distributions were used to build the density plot in Fig EV4F.

C Plot showing the linearity between the pulse-width modulation (PWM) value that controlled LED intensity and the corresponding light output on the optoPlate, which was used to calibrate the optoPlate in subsequent experiments.

D Density plot of NFAT-mediated GFP expression (left) or CD69 upregulation (right) after 24 h of activation when OptoCAR-T cell conjugates were continuously illuminated with varying light intensity. Plot is composed of 85 individual histograms.

E Density plot of NFAT-mediated GFP expression as a function of light intensity continuously applied to OptoCAR-T cells, as in (D). The new range of light intensities was selected to better resolve the bistable region in (D). Plot is composed of 96 individual histograms.

© 2021 The Authors
